# Supplementary material for: Estimating a novel stochastic model for within-field disease dynamics of banana bunchy top virus via approximate Bayesian computation
Source: PLoS Comput Biol. 2020 May 18;16(5):e1007878. doi: 10.1371/journal.pcbi.1007878 (PMC7259802; doi:10.1371/journal.pcbi.1007878)
Supplement: S1 Document — Document providing convergence analysis of MCMC algorithm, algorithm choice and sensitivity analysis of ABC-MCMC tolerance parameter. (DOCX) [file pcbi.1007878.s003.docx]

**Supporting Information**

**ABC-MCMC Convergence Analysis**

The trace plots for all parameters in Fig S1 highlight that the ABC-MCMC chain mixes well, indicating that the samples converge to the approximate posterior distribution of parameters quickly.

The autocorrelation plots for all parameters in Fig S2 exhibit a quick decay in correlation along the chain after thinning, suggesting a reasonable effective number of independent samples in the ABC-MCMC chain.

**Choice of ABC Tolerance.** ABC-MCMC is known to be sensitive to the selected tolerance value, as low tolerance values lead to poor mixing, while excessively high values result in poor approximations of the posterior distributions of the parameters [1]. In order to validate the tolerance value chosen for running our ABC-MCMC algorithm, we may estimate the approximate posterior distribution at lower tolerances by removing accepted ABC-MCMC samples that exceed the designated tolerance at each tolerance threshold from the existing posterior distributions of parameters. Fig S3 provides an indication of the changes in parameter values as the tolerance values decrease.

Fig S3 shows that the parameter values across a small range of tolerance thresholds are largely similar, with minimal changes to the confidence bounds of the approximate posteriors for all parameters. Given these results, and to ensure the computational burden is not too heavy, we decide to keep with our initial choice of tolerance.

**Choice of ABC algorithm.** In this paper we used an MCMC implementation of ABC, but we note that other algorithms could be used, such as sequential Monte Carlo ABC (ABC-SMC) [2, 3].  ABC-SMC traverses a population of parameter samples through a sequence of approximate posteriors with a decreasing ABC tolerance until the desired tolerance is reached or that further reducing the tolerance becomes too computationally demanding.  The advantages of ABC-SMC is that it can exploit the population of parameter samples to adaptively update the proposal distribution that generates new candidate parameter values, run many of the computations on each parameter sample independently on parallel computing devices and it has a better chance of capturing multi-modal posteriors when they occur.  However, it does require sampling a sequence of ABC posteriors, whereas ABC-MCMC directly targets the desired ABC tolerance.  We tried the ABC-SMC approach of Drovandi et al. [3] on our application but found with the vague priors chosen that for relatively large values of the ABC tolerance, the ABC posterior was irregular and the SMC algorithm found it difficult to generate acceptable new candidate parameter values, meaning that ABC-SMC was slow at progressing to smaller values of the ABC tolerance.  However, if we adjust the priors to be uniform with bounds [0, 0.5] rather than [0, 1] bounds, then the sequence of ABC posteriors is substantially more regular, and the ABC-SMC is able to progress to smaller tolerances.  In this case, using the same final target ABC tolerance, we found that the ABC-SMC posterior approximations agreed with ABC-MCMC, providing further validation of our results.

**References**

1. Wegmann D, Leuenberger C, Excoffier L. Efficient approximate Bayesian computation coupled with Markov chain Monte Carlo without likelihood. Genetics. 2009;182(4):1207-18.

2. Sisson SA, Fan Y, Tanaka MM. Sequential Monte Carlo without likelihoods. Proceedings of the National Academy of Sciences. 2007;104(6):1760-5.

3. Drovandi CC, Pettitt AN. Estimation of Parameters for Macroparasite Population Evolution Using Approximate Bayesian Computation. Biometrics. 2011;67(1):225-33.
