# Supplementary figures and images for: Estimating a novel stochastic model for within-field disease dynamics of banana bunchy top virus via approximate Bayesian computation
Source: PLoS Comput Biol. 2020 May 18;16(5):e1007878. doi: 10.1371/journal.pcbi.1007878 (PMC7259802; doi:10.1371/journal.pcbi.1007878)

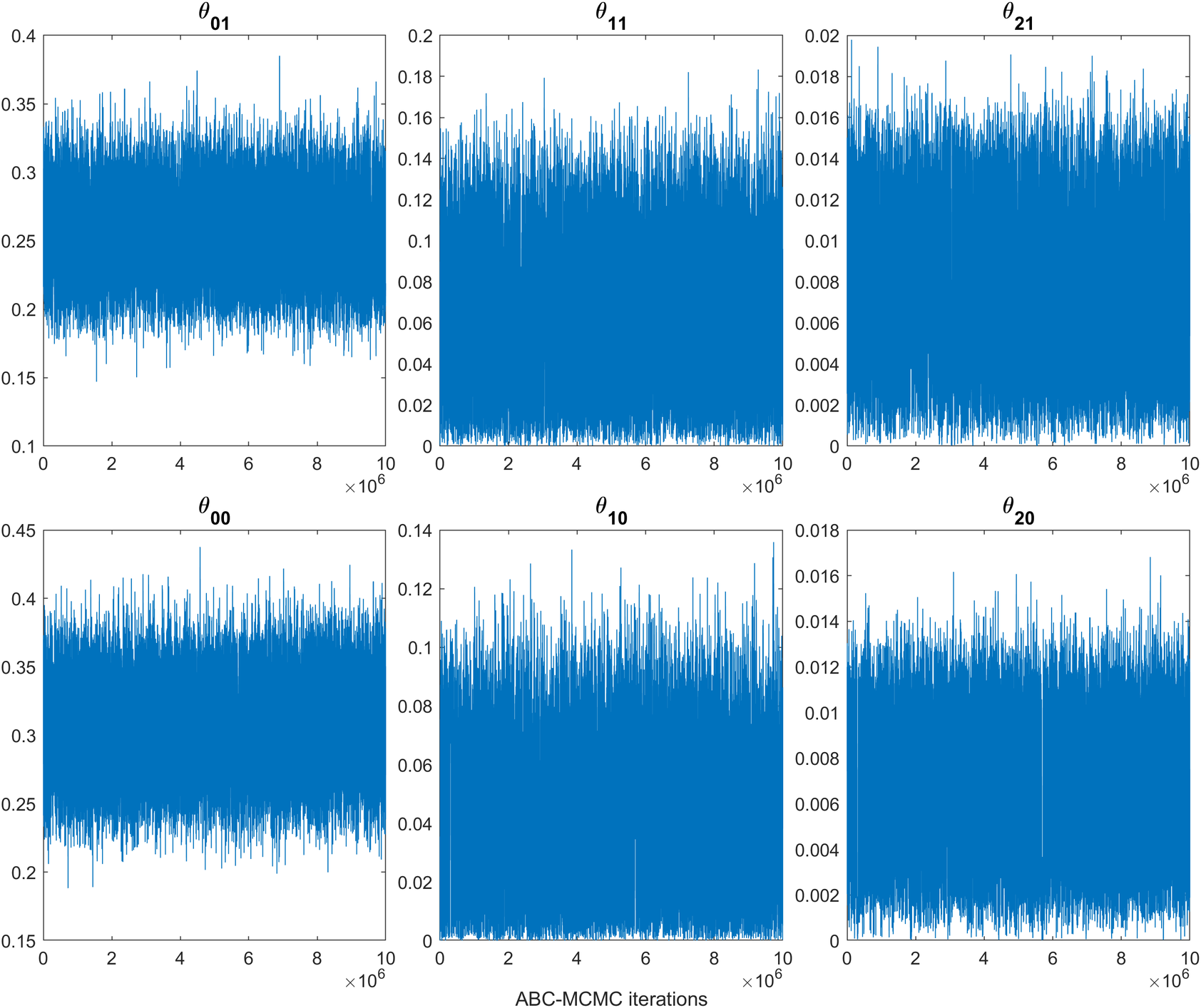

Supplement: S1 Fig — Referred to in S1 Document. (TIFF) [file pcbi.1007878.s004.tiff]

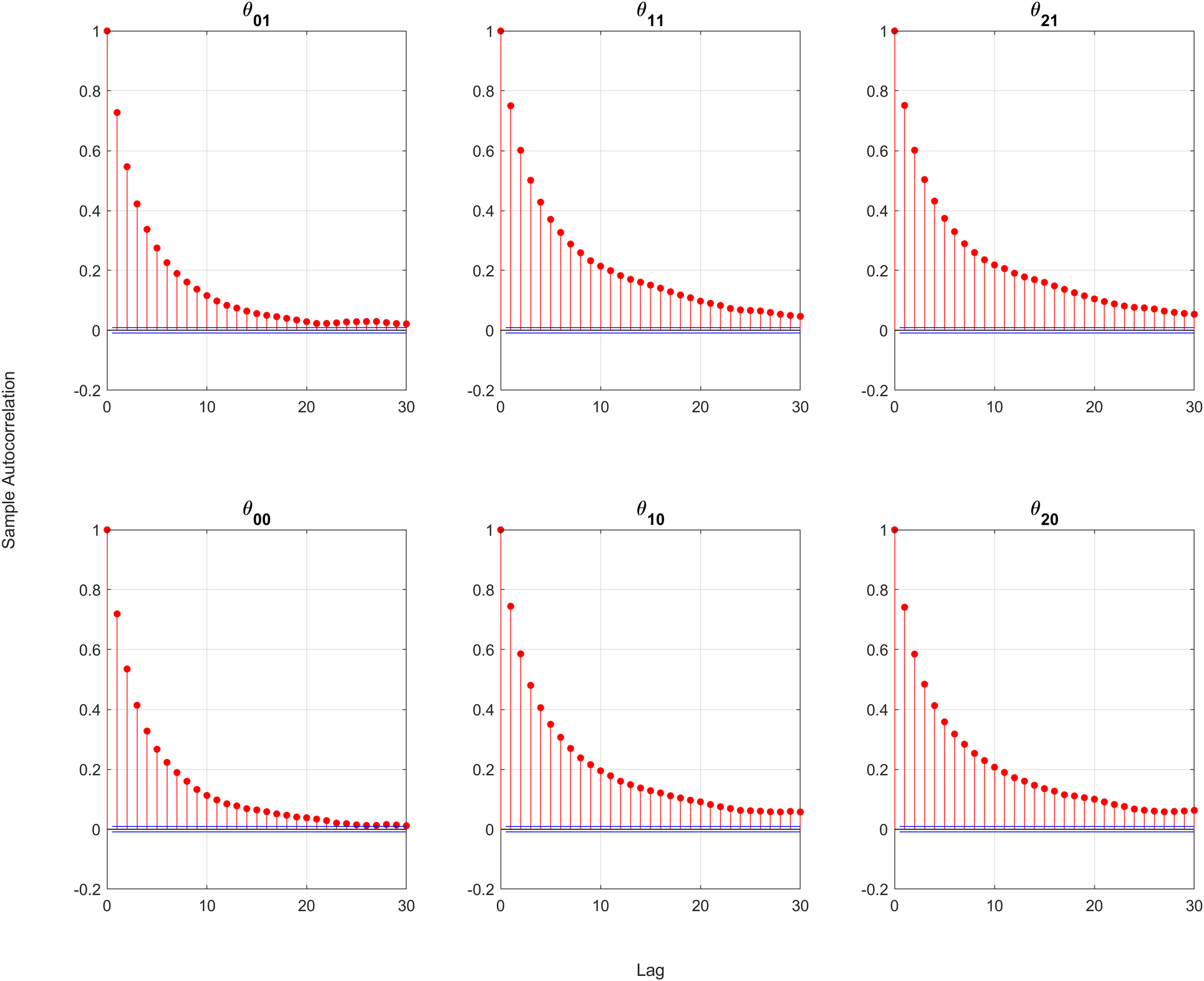

Supplement: S2 Fig — Referred to in S1 Document. (TIFF) [file pcbi.1007878.s005.tiff]

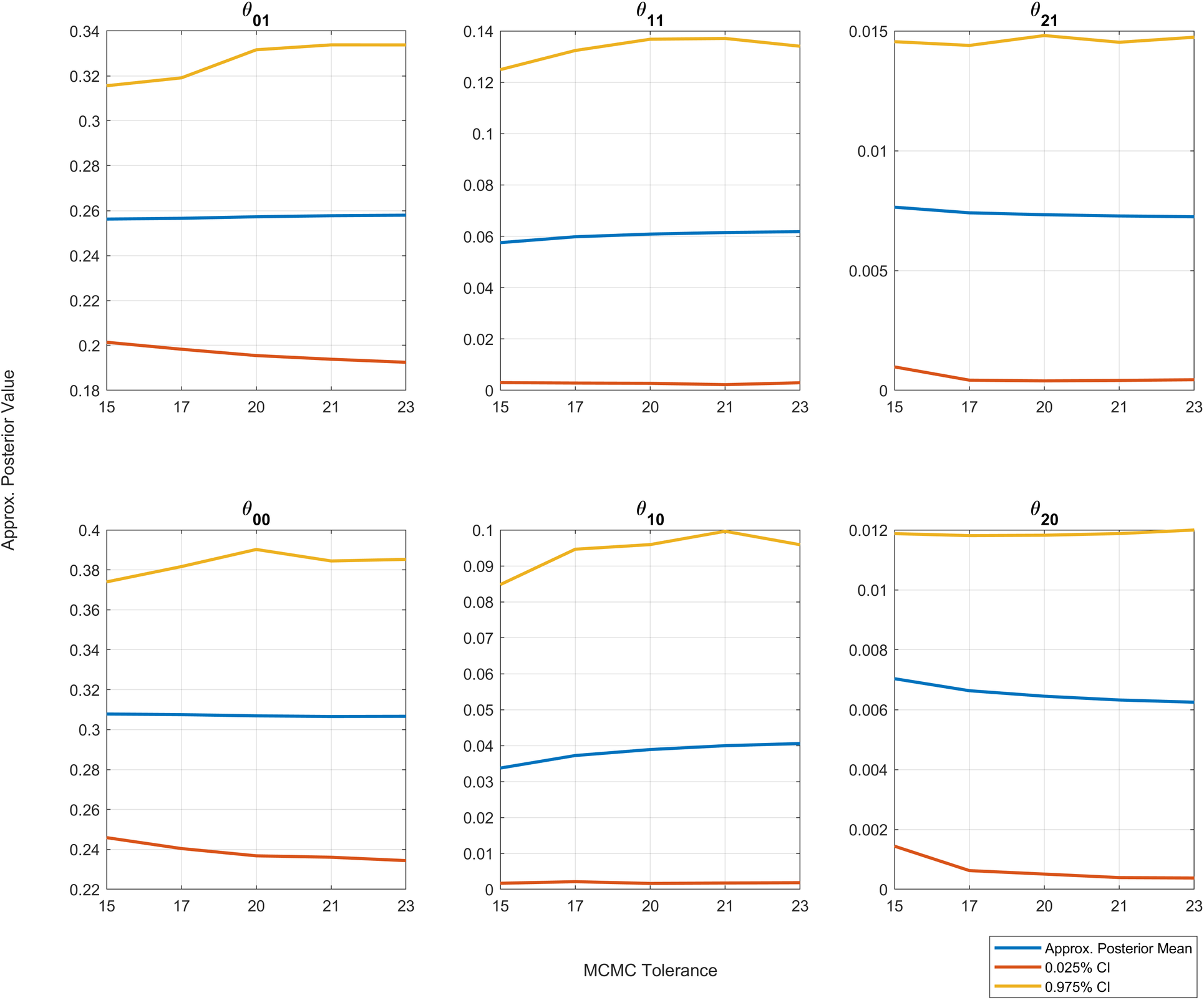

Supplement: S3 Fig — Referred to in S1 Document. (TIFF) [file pcbi.1007878.s006.tiff]
