# Supplementary material for: Estimating a novel stochastic model for within-field disease dynamics of banana bunchy top virus via approximate Bayesian computation
Source: PLoS Comput Biol. 2020 May 18;16(5):e1007878. doi: 10.1371/journal.pcbi.1007878 (PMC7259802; doi:10.1371/journal.pcbi.1007878)
Supplement: Attachment — Submitted filename: Review_PCOMPBIOL-D-19-01505.pdf [file pcbi.1007878.s007.pdf]

Referee's report: PCOMPBIOL-D-19-015-05

Estimating a novel stochastic model for within-field disease dynamics of banana bunchy top virus via approximte Bayesian computation

Authors: Varghese, Drovandi, Mengersen and Mira

This paper studies an $S \rightarrow I$ epidemic amongst banana trees. There are similarities in the model to earlier work presented in [1, 2, 3, 4, 5] where $S \rightarrow I$ epidemics for plants have been considered. The previous works mentioned have focussed on using continuous time models and MCMC to estimate the parameters driving the disease model. In contrast the current manuscript uses a discrete time model and ABC (approximate Bayesian computation) to estimate the model parameters. It would be good for the authors to review their work in light of [1, 2, 3, 4, 5], in particular, I find it difficult to see why an MCMC algorithm has not been used in the current model. It appears, unless I am missing something, that a complete snapshot of the infectious status of the plantation is taken at each timepoint. That is, the infectious status of each individual in the population is known. Therefore it is straightforward to write down the likelihood for the transition of the infectious status of individuals from one time point to the next. In such circumstances implementing MCMC should be trivial and more reliable than ABC.

Despite the query regarding whether ABC is the right approach to this problem, the use of ABC methods for epidemics is an important research area. I would like to see something said about the choice of MCMC-ABC rather than SMC-ABC, although the expected uni-modality of the parameters makes this a reasonable approach.

References

[1] Brown, P.E, Chimard, F., Remorov, A., Rosenthal, J.S. and Wang, X. (2014) Statistical Inference and Computational Efficiency for Spatial Infectious-Disease Models with Plantation Data. *J. of Royal Stat. Soc., Series C* **63**, 467–482.

[2] Gibson, G.J. (1997a) Markov Chain Monte Carlo Methods for Fitting Spatiotemporal Stochastic Models in Plant Epidemiology. *J. of Royal Stat. Soc., Series C* **46**, 215–233.

[3] Gibson, G.J. (1997b) Investigating mechanisms of spatiotemporal epidemic spread using stochastic models. *Amer. Phytopathological Soc.* **87**, 139–146.

[4] Gibson, G.J., Otten, W., Filipe, J.A.N., Cook, A., Marion, G. and Gilligan, C.A. (2006) Bayesian estimation for percolation models of disease spread in plant populations. *Stat. Comput.* **16**, 391–402.

[5] Neal, P. and Xiang, F. (2017) Collapsing of non-centered parameterised MCMC algorithms with applications to epidemic models. *Scan. J. Statist.* **44**, 81–96.
