# Supplementary material for: Estimating a novel stochastic model for within-field disease dynamics of banana bunchy top virus via approximate Bayesian computation
Source: PLoS Comput Biol. 2020 May 18;16(5):e1007878. doi: 10.1371/journal.pcbi.1007878 (PMC7259802; doi:10.1371/journal.pcbi.1007878)
Supplement: Attachment — Submitted filename: Response to Reviewers_Submission.docx [file pcbi.1007878.s008.docx]

**Response to Reviewers**

12 Dec. 19

We would like to thank the reviewers for the time to review this manuscript and for their helpful comments and suggestions. Responses to the comments and a description of revisions to the manuscript have been given below.

**Response to Reviewer #1:**

1. *It is not clear what the network is. How many nodes, how many links, what is the degree distribution? Is this a fully spatial network, apart from the long-range transmission? Do patches separated by dirt tracks define nodes? Are all adjacent patches considered as neighbours? Would be useful to see some summary statistics of this spatial network.*

We thank the reviewer for these queries. While we describe the network and define its nodes and links in lines 119-134 in the original manuscript, we appreciate the need to clearly outline the network characteristics before proceeding to describe the network model. Therefore, we have included a visualisation of the spatial network representing the banana plantation along with its corresponding degree distribution in Fig 3 – which should complement the description of the network characteristics provided in lines 138-150. Further summary statistics of the network has been provided in the caption for Fig 3.

2. *Data on which the parameter estimation has been done is never presented, plotted or analysed. I would expect that at least some of the paper presents and analyses this data.*

We thank the reviewer for raising this concern. We have included the time series plots of the infection counts and the log of the infection counts in Fig. 2 in the revised manuscript to provide a clear understanding of the disease spread and included some further information on the temporal disease characteristics through lines 109-120.

Additionally, we have provided visualisations of the spatial distribution of the BBTV infections over the entire survey period through Fig 4 to further inform readers, and complement the discussion on the development of our network model (lines 134-150).

3. *Why are there no comparisons between the true data and model output? Would like to see how output from the models with the best parameters compares to the true data.*

The section on posterior predictive checking initially present in the Supplementary Information in the original manuscript is dedicated to comparing the performance of model predictions to a validation dataset, having estimated parameters based on a training dataset. In this section, the performance is evaluated through a binomial deviance loss function, which evaluates the deviance between a node’s predicted probability of infection at time *(t+1)* to the true node infection state at time (*t+1)*. Secondly, the evaluation of a Reciever-Operator-Characteristic (ROC) Curve for our model using the validation dataset highlights the change in true- and false-positive rates, as the posterior predictive threshold increases. Therefore, we believe that the inclusion of this section to the main paper is sufficient to address this query, and inform readers on the performance of our model to generate predictions of the future spread of BBTV within a plantation.

4. *The simulation seems to be a discrete time simulation with an arbitrary update windows, so how do you compare abstract time to real time that it is present in the real data? Is the abstract time-step set to some real time like day or week or so on? It is never mentioned over what time window has the plantation been observed and some plots of how the number of infected patches evolved in time.*

*See my comment about the lack of analysis of the data on which the inference is based on.*

We thank the reviewer for raising this concern. Regarding the discrete time simulations, lines 98-102 in the original manuscript provides an indication that the field data is collected at monthly intervals. However, we appreciate the importance of clarity here, and have taken steps to incorporate these changes in the revised manuscript. The revised version of the manuscript clarifies the frequency of field data collection in line 101-103. Since the field data is collected at monthly intervals, our network model also operates on discrete monthly time intervals.

The time window observed is clearly detailed in line 103 of the revised manuscript, which records a time window of December 2014, to January 2018.

Fig 4(c) in the revised manuscript provides an indication of the number of subsection/nodes with at least 1 infection at each time step over the observation period.

5. *At some point the authors talk about SI model but their model is SIS.*

We thank the reviewer for identifying this error. All instances of this have been fixed in the revised manuscript.

6. *What is the initial condition for the simulation?*

We thank the reviewer for raising this query. The initial condition is described in line 134 of the original manuscript and has been rewritten for greater clarity in line 157 of the revised manuscript.

7. *Nodes are sometimes referred to as patches then subsections and other versions, can there be some consistency?*

We thank the reviewer for raising this issue and have revised the manuscript to be more consistent in its terminology. While the original manuscript never uses the word “patches” or any other variations, it does often interchange between “subsections” and “nodes” when referring to the contiguous spaces in the banana plantation and their corresponding nodes in our network model. In the revised manuscript, the term “subsection” is used to directly refer to these spatial regions in the plantation, while the term “nodes” have been used in context of our network model. In rare cases where both terms might be suitable, the term “node/subsection” has been utilized.

8. *Supplementary material in my opinion is more important than some of the figures in the paper. These essentially try to make a case that the model is good/valid. I would move this to the main part of the paper and would make a better case that the model is valid by comparing model output to existing data over the whole period of collection. Or by devising a test whereby one divides the existing data in “used for inference” and “used for validation” and tests performed over different such divisions to validate the model properly.*

We thank the reviewer for providing the suggestion to incorporate the Supplementary Information to the paper. We have added the section on ‘posterior predictive checking’ to the main part of the paper (lines 518 – 557).

In regard to providing a case for model validity, we believe the section on ‘posterior predictive checking’ covers this quite well. In this section, we utilise an additional 7 months of survey data which provides us 7 individual validation datasets to validate the model (where time *t* of the validation dataset month is set as the initial configuration, and time *t+1* is predicted). This method is perhaps a better test of model validity as opposed to utilizing existing data to validate model predictions since this validation data set has not been used for model parameter estimation, which means that the model cannot rely on any embedded information in the estimated parameters to create predictions for (*t+1*).

9. *So the role of the vector is simply the long-range infection?*

We thank the reviewer for this query. In the revised manuscript, we clarify that the aphid vector plays the primary role in both the short-distance and long-range infection (line 186).

10. *I am not sure if the choice of statistics is the best for comparing data to simulation.
    First, I would like to see the data. Is it simply, time, patch, status? Is there seasonality in the collected data?*

We thank the reviewer for raising this concern. Having addressed queries regarding the visualisation and preliminary analysis of the field data in pts 1, 2, and 4, we have also added some further discussion on the development of our summary statistics and justified its reasonableness in lines 303-338 in joint request by Reviewer 2.

11. *For reproducibility purposes is the code available on Github? What about the data?*

Thank you for the suggestion. While both the code and data used were provided as supplementary information in the original manuscript submission, we have also uploaded the data and the code to a public repository in GitHub. Although only the files are present at the moment for download, we hope to provide further support for practitioners to use our code by including examples and documentation on this repository page in the near future. The public repository is available at: [www.github.com/sheksta/bbtv-abc](http://www.github.com/sheksta/bbtv-abc) .

**Minor comments addressed from R1:**

1. ***Line 114:*** *over a range of plantation*

We thank the referee for the correction, this has been addressed in line 128 of the revised manuscript.

2. ***Line 132-133:*** *what do the authors mean by “the new BBTV
   model does not estimate for an initial seed node”? Do they mean that their model is not able to infer the initial seed?*

The referee is correct. This has been clarified in line 162-164 of the revised manuscript.

3. ***Line 191:*** *I think it should be /pi(\phi) not \phi(x) in the integral*

We thank the referee for the correction, this has been addressed in line 227 of the revised manuscript.

4. ***Line 315:*** *In the sentence “Fig 6 provides a posterior infectivity forecast for all subsections over a 6-month period” what does “subsections” refer to? Is this each node/patch?*

We thank the referee for the correction, this has been addressed in line 456 of the revised manuscript.

5. ***Line 363-364:*** *“if subsections 20 to 34 (in grey) were cleared” – is this equivalent o removing nodes?*

We thank the referee for the query, clearing subsections is equivalent to freezing these nodes in a susceptible state. Therefore, the model can propose these nodes for long-range or short-range transmission, but it will never successfully infect that node. This has been clarified in lines 502-504 of the revised manuscript.

6. ***Line 408-410:*** *again this is not an SI model, in epidemiology this is an SIS model.*

We thank the referee for the correction, this has been addressed throughout the revised manuscript.

7. ***Line 468-472:*** *what ranges of values can formula 2 take and what value means good fit versus bad fit?*

We thank the referee for the correction, this has been addressed in line 533-535 of the revised manuscript.

**Response to Reviewer #2:**

1. *This paper describes the development and fitting of a stochastic SIS model of banana bunchy top virus (BBTV) to some novel data from a plantation in Australia. The model is fitted to the available data using ABC-MCMC. The underlying disease dynamics involve an intermediate aphid vector, however data on aphid populations are unknown, and instead the authors simplify the underlying model to look at aggregate probabilities of localised transmission and long-range transmission between “nodes” in a network, where each node corresponds to a group of plants in spatially contiguous areas. This approach has the advantage of not having to model the aphid vector dynamics, at the cost of modelling at quite a course spatial level. They extend their model to use different transmission and recovery probabilities in different seasons (summer/winter) in order to try to assess the impact of weather / temperature etc. on the transmission dynamics.

   The results suggest some marginal impact of season on localised transmission potential, and negligible impact of season on long-range transmission and recovery.*

*Overall I think the paper looks fine. It’s a relatively straightforward application of ABC methodology to a novel data set, and would be of interest to the readers of the journal call.*

We thank the reviewer for their positive comments, and for a great summary of the motivations and results of our network model.

2. *The authors state a “significant” impact of season on localised transmission potential, but I think that the approximate posteriors overlap a lot here and so I might argue that the effect is not that “significant”.*

We thank the reviewer for raising this concern. The term “significant” has been removed when referring the impact of seasonality on localized transmission potential.

3. *In my opinion there also needs to be more discussion of the ABC tolerance used in the ABC-MCMC, and what impact that might have made on the results as well. Perhaps subtle differences are being masked by the approximations introduced in the ABC, and this warrants further discussion.*

We thank the reviewer for providing this suggestion. Lines 351-354 of the revised manuscript now detail the ABC tolerance value used for generating the approximate posteriors for our model parameters. A section on ABC-MCMC convergence analysis has also been added, where lines 422-436 discuss the influence of changes to the ABC tolerance on the posterior distributions of the parameters.

4. *Another discussion point could be whether the use of a combined metric might be averaging out some of the information in the data.*

We thank the reviewer for raising this concern. Assuming that the reviewer is referring to our use of several scalar summary statistics summed over all time steps as opposed to a vector with each time step, we have provided a justification for this in the revised manuscript (lines 324-338).

However, if the reviewer is referring to the choice of the combined metric for the discrepancy measure used in the ABC-MCMC algorithm, it must be noted that ABC algorithms generally require a scalar discrepancy measure to estimate the posterior distributions of the parameters.

**Minor comments addressed from R2:**

1. ***Abstract (and elsewhere):*** *I think that technically the authors are using an SIS model, not an SI model per se, because there are transitions back to the S class (and hence why they observe endemic equilibriums in their forecasts).*

We thank the referee for the correction, this has been addressed in throughout the revised manuscript.

2. ***L57, L94:*** *Sometimes there are capital letters in e.g. “North-Eastern”* ***(L94)*** *but other times lower-case letters e.g. “south-east”* ***(L57)****. These should be consistent throughout the manuscript.*

We thank the referee for the correction, this has been addressed in the revised manuscript.

3. ***L128:*** *Sentence beginning, “Secondly, ….” does not read well. Suggest a re-write to clarify what the authors mean.*

We thank the referee for the correction, this has been addressed in line 159 of the revised manuscript.

4. ***L144:*** *“rogueing” should be “roguing”.*

We thank the referee for the correction, this has been addressed in line 183 of the revised manuscript.

5. ***L160:*** *The figure citations in the text are all out by one (and throughout the rest of the manuscript). This line should read “Fig. 1” and not “Fig. 2”, and this carries through the paper.*

We thank the referee for the correction, this has been addressed in the revised manuscript.

6. ***Equation 4:*** *Should this have a 1/n outside the sum?*

We thank the referee for the correction, this has been addressed in the revised manuscript.

7. ***L242:*** *Some more exposition about how the authors figured out which summary measures were informative would be interesting. What criteria did they use?*

We thank the referee for the suggestion, the discussion on the summary statistics has been extended in lines 303-338 of the revised manuscript.

8. ***L245:*** *It might be worth clarifying in the notation that S1 is specified for each time point (all the other summary measures are explicitly stated to be at time point t, except the first).*

We thank the referee for the correction, this has been addressed in line 296 of the revised manuscript.

9. ***P13:*** *How many replicates simulations were used to generate the estimate in equation 4? This should be stated somewhere.*

We thank the referee for the correction, a section on the ‘number of MCMC iterations’ has been incorporated in lines 346-350 of the revised manuscript.

10. ***Fig 4:*** *It looks like the plot in the bottom-right has had its axes flipped compared to the other plots. Is this correct?*

We thank the referee for the correction, this has been addressed in the revised manuscript.

11. ***P21:*** *Why did the authors choose to remove the grey squares? Why these and not any other squares? Possibly Fig 8 might be more informative if the differences were mapped, rather than the absolute values.*

We thank the referee for this suggestion. We have added information on the motivation for removing the grey squares in lines 505-507 of the revised manuscript. Furthermore, we have revised the figure (Fig 14 in the revised manuscript) with the differences between Fig 13 mapped instead in lines 508-517 of the revised manuscript.

12. ***L384:*** *“...their priori...” doesn’t make sense.*

We thank the referee for the correction, this has been addressed in line 566 of the revised manuscript.

13. ***L411:*** *Here the authors use “MCMC-ABC” but elsewhere they use “ABC-MCMC”.*

We thank the referee for the correction, this term has been made consistent in the revised manuscript and we have chosen to use “ABC-MCMC”.

**Response to Reviewer #3:**

1. *It would be good for the authors to review their work in light of [1, 2, 3, 4, 5], in particular, I find it difficult to see why an MCMC algorithm has not been used in the current model. It appears, unless I am missing something, that a complete snapshot of the infectious status of the plantation is taken at each timepoint. That is, the infectious status of each individual in the population is known. Therefore, it is straightforward to write down the likelihood for the transition of the infectious status of individuals from one time point to the next. In such circumstances implementing MCMC should be trivial and more reliable than ABC. Despite the query regarding whether ABC is the right approach to this problem, the use of ABC methods for epidemics is an important research area.*

We thank the reviewer for their comments. It is important to clarify that while the continuous-time models described in [1,2,3,4,5] are used in very similar applications to our modelling problem, our use of a network model to describe the disease dynamics within a banana plantation is a significant distinguishing factor. It is the use of a complex network structure to define the spatial relationships between the plantation subsections that creates significant challenges to derive the likelihood function for this model. Therefore, given the low computational expense to simulate, we turn to ABC to approximate the posterior distributions of the parameters for this model. We believe that providing readers with a comprehensive view of the modelling approaches to this problem, in additional to clearly elucidating the motivation for ABC is important. Therefore, we have included a discussion on the motivation for ABC in lines 231-250 in the revised manuscript, in addition to recognizing alternative modelling approaches where likelihood-based inference methods such as MCMC may be used.

2. *I would like to see something said about the choice of MCMC-ABC rather than SMC-ABC, although the expected uni-modality of the parameters makes this a reasonable approach.*

We thank the reviewer for pointing this out. A discussion on the choice of the ABC Algorithm has been incorporated in lines 437-454 in the revised manuscript.

**References in Reviewer 3’s comments:**

[1] Brown, P.E, Chimard, F., Remorov, A., Rosenthal, J.S. and Wang, X. (2014) Statistical Inference and Computational Efficiency for Spatial Infectious-Disease Models with Plantation Data. J. of Royal Stat. Soc., Series C 63, 467-482.

[2] Gibson, G.J. (1997a) Markov Chain Monte Carlo Methods for Fitting Spatiotemporal Stochastic Models in Plant Epidemiology. J. of Royal Stat. Soc., Series C 46, 215-233.

[3] Gibson, G.J. (1997b) Investigating mechanisms of spatiotemporal epidemic spread using stochastic models. Amer. Phytopathological Soc. 87, 139-146.

[4] Gibson, G.J., Otten, W., Filipe, J.A.N., Cook, A., Marion, G. and Gilligan, C.A. (2006) Bayesian estimation for percolation models of disease spread in plant populations. Stat. Comput. 16, 391-402

[5] Neal, P. and Xiang, F. (2017) Collapsing of non-centered parameterised MCMC algorithms with applications to epidemic models. Scan. J. Statist. 44, 81-96
