# Supplementary material for: Estimating a novel stochastic model for within-field disease dynamics of banana bunchy top virus via approximate Bayesian computation
Source: PLoS Comput Biol. 2020 May 18;16(5):e1007878. doi: 10.1371/journal.pcbi.1007878 (PMC7259802; doi:10.1371/journal.pcbi.1007878)
Supplement: Attachment — Submitted filename: Response to Reviewers_v2.docx [file pcbi.1007878.s009.docx]

**Response to Reviewers**

4 Apr. 20

We would like to all reviewers for taking the time to review this manuscript and providing helpful comments and suggestions. Responses to the comments and a description of revisions to the manuscript have been given below.

**Response to Reviewer #2:**

1. *This is a review of a revised version of the above manuscript. I thank the authors for taking the time to consider and act on the comments from the first review. In my opinion, the manuscript is now much improved and clearer as to the intent, novelty and limitations of the study. As such I think it is suitable for publication, subject to a few minor amends as highlighted below.*

We thank the reviewer for their positive comments, and useful feedback during the revision phase of this manuscript.

2. *One point is that there are now a lot of figures. I appreciate that the authors have moved some figures from the Supplementary Information to the main paper in response to some comments from the other reviewers. However, I think the balance has shifted a bit too far. For example, Fig. 7 is effectively the diagonals of Fig. 8, and as such I would remove Fig. 7 and just reference Fig. 8. I would also put all the MCMC convergence plots into the Supp Mat (e.g. Figs 9 and 10). Also, Fig 11 is a sensitivity analysis type plot and I think it should be in Supp Mat also.*

We thank the reviewer for raising this concern. In accordance with the recommendation provided, we have moved the “MCMC Convergence Analysis” section to the Supplementary Material.

While we understand that Fig. 7 and Fig. 8 utilise the same dataset and are similar, they serve different purposes in this paper. Fig. 7 provides a clear way for the reader to compare the approximate posterior distributions across the model parameters and seasons. Whereas, Fig. 8 seeks instead to highlight pairwise correlation in the approximate posteriors. Given that we feel both figures provide individual value, and serve to increase readability, we have chosen to keep both figures as they are in this paper.

3. *There is also no Fig. 12 as far as I can see, so again, please take care with figure numbering.*

We apologise for this oversight and thank the reviewer for making us aware of the missing figure. The figure was missed while uploading the revised copy.

**Minor comments addressed from R2:**

1. ***Abstract, L33:*** *I don’t think the “predict for” is correct here. Would suggest something like:*

*“Our models can be used to quantify the effects of …. BBTV spread and can be used to predict high-risk areas in this plantation.”*

We thank the referee for the correction, this has been addressed in the revised manuscript.

2. ***L131:*** *Should probably reference the exact GIS software used.*

We thank the referee for the correction, this has been addressed in the revised manuscript.

3. ***L281:*** *Should probably be \rho(S(x), S(y)) to be consistent with previous paragraph applying the distance metric to summary measures.*

We thank the referee for the correction, this has been addressed in the revised manuscript.

4. ***L297-300:*** *Please make it clear that these are summed across all time points. This is not highlighted in the text until the following page, and I think it would help clarity to make this explicit here.*

We thank the referee for the correction, this has been addressed in the revised manuscript.

5. ***Table 1:*** *Should the summer and winter be j = 0 and j = 1 respectively (not j = 1 and j = 2)?*

We thank the referee for the correction, this has been addressed in the revised manuscript.

6. ***L431:*** *This is interesting, but I disagree slightly with the author’s interpretation. To my mind, what they have done is conduct a sensitivity analysis around what would happen to the posterior marginal Cis if they were to change the tolerance. They suggest that since the CIs don’t change much, then the “chosen tolerance value is reasonable for our application”. I might suggest that what they’ve done is assess the relative impacts of small changes, rather than the absolute impacts. I would suggest rewording this to something along the lines of, “small changes to the tolerance do not seem to impact on the approximate posterior uncertainties here, and so we decide to keep with our initial choice of tolerance.”*

*This also relates to my earlier question (apologies for not making this clear in the first review), that a choice of an aggregated discrepancy measure might average out some information in the data (an alternative is to use multiple distance metrics around each summary measure, each with a different tolerances e.g. Ratmann et al. 2009).*

We thank the referee for the suggestion and have reworded the paragraph of the revised manuscript to be accurate (Supporting Information – line 23).

In addition, we have clarified our approach on distance measures for ABC and have referred to potential alternative methods (e.g Ratmann et al., 2009) in line 347 of the revised manuscript.

7. ***L592:*** *“Significant” seasonality?*

We thank the referee for the correction, this has been addressed in the revised manuscript.
